# Guidewire use in electrocautery-enhanced lumen-apposing metal stent procedures: Results of an online survey from an international working group

**DOI:** 10.1055/a-2854-7045

**Published:** 2026-05-20

**Authors:** Tawfik Khoury, Michiel Bronswijk, Wisam Sbeit, Pietro Fusaroli, Rares I. Orzan, Khanh Do-Cong Pham, Sundeep Lakhtakia, Jeremie Jacques, Carlos Robles-Medranda, Anthony Y.B. Teoh, Benedetto Mangiavillano, Giuseppe Vanella, Antonio Facciorusso, Andrea Lisotti, Bertrand Napoleon

**Affiliations:** 1 Department of Gastroenterology 61255 Galilee Medical Center, Bar-Ilan University, SafedNahariyaIsrael; 2 Department of Gastroenterology 89686 Jean Mermoz Private HospitalLyonAuvergne-Rhône-AlpesFrance; 3 Gastroenterology and Hepatology 60182 KU Leuven University Hospitals LeuvenLeuvenBelgium; 4 Department of Gastroenterology and Hepatology 81874 Imelda HospitalBonheidenBelgium; 5 Department of Gastroenterology 61255 Galilee Medical CenterNahariyyaNorth DistrictIsrael; 6 Gastroenterology Unit, Hospital of ImolaUniversity of BolognaImolaItalyItaly; 7 433709 Regional Institute of Gastroenterology and HepatologyCluj-NapocaCJRomania; 8 3rd Department of Internal Medicine 37576 Iuliu Hațieganu University of Medicine and PharmacyCluj-NapocaCJRomania; 9 Department of MedicineHaukeland University HospitalBergenNorway; 10 Medical Gastroenterology 672640 AIG HospitalsHyderabadTelanganaIndia; 11 Service d'Hépato-Gastro-EntérologieCHU Dupuytren LimogesLimogesFrance; 12 GastroenterologyInstituto Ecuatoriano de Enfermedades Digestivas - IECEDGuayaquilGUAYASEcuador; 13 Surgery 13620 Hong Kong Sanatorium & Hospital LimitedHong KongHong Kong; 14 Gastrointestinal Endoscopy UnitHumanitas Mater DominiCastellanzaItaly; 15 Division of Gastroenterology and Digestive Endoscopy 9268 IRCCS Humanitas Research HospitalRozzanoLombardiaItaly; 16 Pancreatobiliary Endoscopy and Endosonography DivisionIRCCS San Raffaele Scientific InstituteMilanItaly; 17 Gastroenterology Unit, Department of Experimental Medicine 18976 Università del SalentoLecceItaly; 18 Gastroenterology Unit, Hospital of Imola 9296 University of BolognaImolaItaly

**Keywords:** guidewire, LAMS, hot axios, hot spaxus, complications

## Abstract

**Background and study aims:**

This international survey aimed to characterize current practice patterns, perceptions, and outcomes related to guidewire use in electrocautery-enhanced lumen-apposing metal stents (EC-LAMS) procedures.

**Methods:**

An online survey of experienced therapeutic endoscopists was conducted, collecting information on preferred techniques, indications for guidewire use, perceived risks, and self-reported adverse events (AEs). Post-hoc analyses explored associations between guidewire use, AEs, and operator experience.

**Results:**

Eighty-seven endoscopists (mean age 46.1 years, 88.5% with ≥ 5 years of endoscopic ultrasound (EUS) experience) completed the survey. Freehand deployment was the preferred technique for the majority of all procedures. Guidewire use was mainly preferred for EUS-guided choledochoduodenostomy (EUS-CDS, 24.1%) and EUS-directed transgastric endoscopic retrograde cholangiopancreatography (EDGE, 31%). Reported factors favoring guidewire use included small target lumens and difficult scope positioning. Maldeployment was reported as the most important complication, because 49.3%, and 40.6% of the participants reported at least one maldeployment case in EUS-CDS and EUS-guided gastroenterostomy, respectively. Logistic regression showed that guidewire use in EUS-CDS was perceived to result in higher complication rates (44.1% vs 20.0%; odds ratio 3.16, 95% confidence interval 1.08–9.2, *P* = 0.03). No significant differences in outcomes or guidewire use were observed by operator experience or case volume, although older endoscopists were more likely to use guidewires.

**Conclusions:**

Using an international questionnaire for experienced EUS operators, freehand deployment was identified as the predominant technique for EC-LAMS placement.

## Introduction

Endoscopic ultrasound (EUS)-guided interventions have greatly expanded endoscopic capabilities, facilitating minimally invasive drainage of intraabdominal fluid collections and luminal bypass procedures 1 . Among the most significant innovations is the development of lumen-apposing metal stents (LAMS), large-caliber, dumbbell-shaped devices engineered to create and maintain secure fistulous tracts 2 . Introduction of electrocautery‑enhanced LAMS (EC‑LAMS) has further revolutionized these procedures. Devices such as the Hot AXIOS (AXIOS; Boston Scientific Medical Co., Marlborough, Massachusetts, United States), Hot Spaxus (Niti-S SPAXUS; Taewoong Medical Co., Goyang, Korea), and Hot-Plumber (HANAROSTENT Hot-Plumber with ZEUS IT; MI Tech Co., Seoul, Korea) integrate a cautery tip into the delivery catheter, allowing direct transmural puncture and stent deployment in a single step. Once the distal flange is deployed within the target lumen, the system retracts to release the proximal flange under endoscopic control. This standardized delivery technique eliminates the need for prior needle puncture, tract dilation, or multiple accessory exchanges, thereby reducing technical complexity and procedure time 3 4 . EC-LAMS are now widely applied for pancreatic fluid collection (PFC) drainage, EUS-guided gallbladder drainage (EUS-GBD) in high-risk surgical patients, EUS-guided choledochoduodenostomy (EUS-CDS) for biliary decompression in distal biliary obstruction, EUS-guided gastroenterostomy (EUS-GE) for gastric outlet obstruction, and EUS-directed transgastric or transenteric ERCP (EDGE/EDEE) in patients with surgically-altered anatomy 1 5 6 .

Despite these advances, an important technical question remains unresolved: Should EC-LAMS be deployed freehand, or over a guidewire? Traditional EUS-guided transmural drainage techniques (before LAMS or with non-cautery devices) nearly always involved a multistep “Seldinger” approach: needle puncture, guidewire placement, tract dilation, and then stent insertion 7 . With the “hot” LAMS systems, freehand single-step deployment has been suggested, a method embraced by the endoscopic community for its efficiency and reduced need for device exchanges 4 8 . However, many endoscopists continue to incorporate a guidewire for each procedure, either as a safety measure to secure access or to maintain access during difficult maneuvers 9 .

Potential advantages of guidewire use include confirmation of luminal access (by contrast injection or fluoroscopy), stabilization of the tract, and as a safety measure in technically demanding cases. Conversely, guidewires increase procedure complexity, requiring accessory exchanges, and may occasionally kink, migrate, or dislodge during device insertion, paradoxically raising risk of maldeployment 6 10 . Despite the theoretical benefits, there is a lack of consensus or formal guidelines on this aspect, and practices vary widely based on training, experience, and personal preference 10 . Even in multicenter trials, guidewire insertion is left to operator discretion 11 .

In this context, we conducted a worldwide international survey of therapeutic endoscopists to characterize practice patterns regarding guidewire use during EC-LAMS procedures across five major indications (PFC, EUS-GBD, EUS-CDS, EUS-GE, and EDGE). We aimed to assess factors driving guidewire use, explore perceived procedure risks and outcomes expressed by the operators, and evaluate whether operator experience may influence these decisions.

## Materials and methods

### Study design and participants

We conducted an international cross-sectional study using an online survey questionnaire using Google Forms targeted at interventional endoscopists. A working group developed the survey focused on LAMS techniques, which was distributed electronically via professional networks and society mailing lists between January 1, 2025 and August 1, 2025. Participation was voluntary and anonymous. The target population was endoscopists who perform EUS-guided therapeutic procedures with LAMS. No personal identifying information was collected; respondents were assured of confidentiality, and implied informed consent was assumed by completion of the survey.

Survey items were formulated by expert consensus within the working group and pilot-tested by a few independent endoscopists for clarity and relevance. Minor refinements were made before broader distribution.

The study was deemed exempt from formal ethics board review because it focused on practitioner experiences and opinions. The questionnaire (designed in English) consisted of both multiple-choice and short-answer items and take approximately 5 minutes to complete.

### Demographics and experience

Age, gender, and years of experience in EUS were queried, including subcategories for experience in EUS-guided fine-needle aspiration/biopsy (FNA/FNB) and EUS-guided drainage procedures. Respondents were also asked to indicate the primary type of electrocautery LAMS device they use in practice (e.g., Hot AXIOS, Hot Spaxus, or both).

### Technique preferences

For each of five LAMS procedure types (EUS-GBD, EUS-CDS, EUS-PFC drainage, EUS-GE, and EDGE), participants were asked whether they typically deploy the stent freehand, with a guidewire, or use both techniques depending on the case. If they reported using a guidewire in any capacity, a follow-up question asked which guidewire technique they employ: 1) a standard EUS-FNA needle to access the target and insert a guidewire, then exchange for the LAMS delivery system (“traditional” multistep approach); 2) use of an electrocautery LAMS delivery catheter to access the target and insert a guidewire through the catheter before deployment (guidewire-assisted one-step approach); or 3) both methods in different circumstances. Participants were presented with potential factors (including target organ size, difficult endoscope positioning, presence of solid debris in a collection, and coagulopathy) and asked to select which, if any, would prompt them to opt for using a guidewire in each type of procedure.

### Incidence of adverse events

The survey inquired about participant personal experience with complications during LAMS placement for each indication. Specifically, it listed major adverse events (AEs) (e.g., bleeding, perforation, stent maldeployment, and “other”) and asked respondents to indicate which of those they had encountered in their practice for each type of procedure. Questions were intended to gauge perceived incidence of complications in the hands of the respondents rather than exact rates from a formal series. Therefore, we reported the percent of participants who experienced at least one case of each AE.

### Opinions and attitudes

Finally, the survey included questions on participant overall stance regarding guidewire use. This included whether they believe a guidewire improves safety, has no impact, or increases risk; whether they recommend routine guidewire use or not; and how frequently they themselves use a guidewire (with answer choices such as “in < 50% of my LAMS cases” vs “in > 50% of cases”).

### Data collection and analysis

Responses were collected through a secure online platform. For multiple-choice questions, respondents could sometimes select more than one applicable answer (e.g., multiple factors influencing guidewire use), as noted in the survey. All survey data were aggregated and analyzed using standard descriptive statistics. Categorical variables (such as percentages of respondents choosing a given technique or complication) were computed and reported as percentages of respondents to that question. For questions not answered by all participants, the number of respondents for that item was used as the denominator.

To explore relationships in the data, we performed additional statistical analyses. We compared the self-reported complication rates between those who use a guidewire and those who do not, for each type of procedure. Because this was based on categorical survey responses (complication experienced vs not, stratified by technique used), we employed logistic regression to estimate odds ratios (ORs) for the association of guidewire use with the occurrence of an AE in each scenario. We similarly examined whether respondent experience level (< 5 years vs ≥ 5 years, as well as < 10 vs ≥ 10 in some analyses) was associated with differences in overall complication occurrence. We assessed whether experience or case volume correlated with likelihood of using a guidewire (dichotomized as uses a guidewire routinely or often vs uses rarely or never). For experience and volume, categories (for instance, performing < 25 LAMS cases per year vs ≥ 25 cases) were compared. In addition, we investigated the effect of respondent age on guidewire usage patterns for each procedure, given an observed trend, by comparing age groups (arbitrarily split at 40, 50, and 60 years in separate analyses) via chi-square or Fisher’s exact tests and logistic regression. Results of regression analyses are presented as ORs with 95% confidence intervals, and *P* values from Wald tests. No adjustments were made for multiple comparisons because these analyses were exploratory and hypothesis-generating. All statistical analyses were performed using misdeployment SPSS version 29 (IBM Corp., Armonk, New York, United States).

## Results

### Participant demographics and experience

A total of 87 endoscopists completed the survey. Baseline characteristics of the respondents are summarized in Table 1 . Mean age of participants was 46.1 years (SD ± 9.6, range 30–68), and most were male (74/87, 85.1%). Almost half of the participants were from France (n = 39), followed by Italy (n = 13), Belgium (n = 7), the United States (n = 6), and other countries ( **Supplementary Fig. 1** ). The cohort was highly experienced, with 88.5% having performed EUS for ≥ 5 years, including 36.8% with ≥ 15 years of EUS experience. Similarly, 89.9% had ≥ 5 years of experience in EUS-guided FNA/FNB (with one-third having > 15 years), and 75.4% in therapeutic EUS procedures such as transmural drainage.

**Table:** **Table 1** Baseline characteristics of the study group.

**Number of participants**	**87**	**Number of participants who answered the question**
Age, mean ± SD (years)	46.1 ± 9.6	87
**Gender, n (%)**		87
Male	74 (85.1)	
Female	13 (14.9)	
**Years of experience in EUS, n (%)**		87
< 5 years	10 (11.5)	
5 to < 10 years	28 (32.2)	
10–15 years	17 (19.5)	
> 15 years	32 (36.8)	
**Years of experience in EUS-FNA/FNB**		69
< 5 years	7 (10.1)	
5 to < 10 years	20 (29)	
10–15 years	19 (27.5)	
> 15 years	23 (33.4)	
**Years of experience in EUS-guided drainage**		69
< 5 years	17 (24.6)	
5 to < 10 years	18 (26.1)	
10–15 years	18 (26.1)	
> 15 years	16 (23.2)	
**LAMS type used, n (%)**		87
Hot Axios	76 (87.4)	
Hot Spaxus	1 (1.1)	
Both	10 (11.5)	
EUS, endoscopic ultrasound; EUS-FNA, EUS-guided fine-needle aspiration; EUS-FNB, EUS-guided fine-needle biopsy; LAMS, lumen apposing metal stent; SD, standard deviation.		

Regarding device use, 87.4% reported the Hot AXIOS electrocautery-enhanced LAMS as their primary system. Only one respondent (1.1%) used the Hot Spaxus LAMS exclusively, whereas 11.5% had substantial experience with both devices.

### Preferred techniques: Freehand vs guidewire in LAMS deployment

For each major application of electrocautery-enhanced LAMS, participants indicated whether they typically deploy the stent freehand, with a guidewire, or variably depending on the case ( Table 2 ). Across all types of procedures, freehand deployment was the predominant approach. A large majority reported always using freehand LAMS placement in PFC drainage (87.4%), EUS-GE (82.8%), EUS-GBD (79.3%), EDGE (66.7%), and EUS-CDS (65.5%).

**Table:** **Table 2** EUS-guided drainage technique.

	**Freehand, %**	**Using guidewire, %**	**Both, %**	**If using guidewire, %**		
				**FNA needle-guidewire-LAMS over guidewire**	**LAMS-preloaded guidewire into target organ**	**Both**
EUS-GBD	79.3%	4.6%	16.1%	13.2%	76.3%	10.5%
EUS-CDS	65.5%	24.1%	10.3%	24.5%	69.8%	5.7%
EUS-PFC	87.4%	4.6%	8%	15.2%	72.7%	12.1%
EUS-GE	82.8%	16.1%	1.1%	17.2%	72.5%	10.3%
EDGE	66.7%	31%	2.3%	67.6%	29.5%	2.9%
EDGE, EUS-directed trans-gastric endoscopic retrograde cholangiopancreatography; EUS-CDS, EUS-guided choledochoduodenostomy; EUS-GBD, EUS-guided gallbladder drainage; EUS-GE, EUS-guided gastroenterostomy; EUS-PFC, EUS-guided pancreatic fluid collection drainage; FNA, fine-needle aspiration; LAMS, lumen apposing metal stent.						

Routine guidewire use was uncommon. Only 4.6% always used a guidewire for EUS-GBD and 4.6% for PFC drainage, whereas one respondent (1.1%) did so for EUS-GE. In contrast, two procedures stood out with higher routine guidewire usage: EUS-CDS (24.1%) and EDGE (31%).

Aside from those who adhere strictly to either freehand or wire-assisted deployment, a minority indicated a mixed approach, most often in EUS-GBD (16.1%), EUS-CDS (10%), and PFC drainage (8%). Very few varied their approach in EUS-GE (1%) or EDGE (2%).

Respondents who used guidewires described using mostly the technique of advancing a guidewire through the electrocautery catheter after target access ( Table 2 , right columns).

In EUS-GBD, 76.3% advanced the wire through the LAMS catheter, whereas 13.2% used the needle-first approach; similar patterns were reported for EUS-CDS (69.8% vs 24.5%), EUS-GE (72.5% vs 17.2%), and PFC drainage (72.7% vs 15.2%). EDGE was the exception: among those who used a guidewire, 67.6% favored the traditional needle-and-wire technique, compared with 29.5% who advanced the wire through the LAMS catheter.

### Factors influencing the decision to use a guidewire

**Fig. 1:**
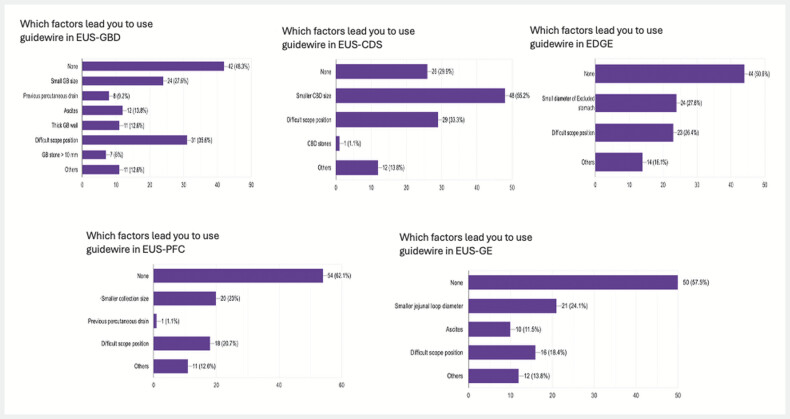
Factors influencing endoscopist decision to use a guidewire in EC-LAMS deployment across different procedure types. EUS-GBD, endoscopic ultrasound-guided gallbladder drainage; EUS-CDS, EUS-guided choledochoduodenostomy; EUS-PFC, EUS-guided pancreatic fluid collection drainage; EUS-GE, EUS-guided gastroenterostomy; EDGE, EUS-directed transgastric endoscopic retrograde cholangiopancreatography.

Respondents were asked under what circumstances they would intentionally use a guidewire instead of deploying a LAMS freehand ( Fig. 1 ). For most procedures, the largest proportion selected “none”: 48.3% for EUS-GBD, 62.1% for PFC drainage, 57.5% for EUS-GE, and 50.6% for EDGE. In EUS-GBD, 35.6% reported using a guidewire for a challenging scope position and 27.6% for a small gallbladder. In PFC drainage, ~20% would use a guidewire in cases of a small collection or difficult scope position. EUS-GE showed a similar pattern: Whereas 57.5% reported no special criteria for using a wire, 24.1% use a guidewire for a small-caliber jejunal loop and 18.4% for a difficult scope position. For EUS-CDS, a small common bile duct (CBD) was the main factor prompting guidewire use, with scope position and large stone burden reported less frequently. In EDGE, 50.6% reported no specific factor prompting use of a guidewire, 27.6% used a wire for a small, excluded stomach, 26.4% for a difficult scope position, and 16.1% for other reasons.

### Adverse events during LAMS procedures

AE data were available from 69 respondents. **Supplementary Table 1** presents self-reported distribution of various complications (bleeding, perforation, maldeployment, and other) for each type of procedure. In EUS-GBD, 58% reported no complications, whereas 42% had encountered at least one AE. Maldeployment was the most common, reported AE by 34.8% of the participants. In EUS-CDS, 59.4% reported at least one AE. Maldeployment was reported by nearly half of respondents (49.3%). For PFC drainage, most respondents reported no complications. Among those who did, bleeding was the most common, reported by 26.1% of the participants. In EUS-GE, 52.2% reported at least one complication. Of the participants, 40.6% reported maldeployment and 11.6% reported perforation. For EDGE, 13% of participants reported maldeployment and 7.2% reported bleeding.

### Participant opinions on guidewire use

**Fig. 2:**
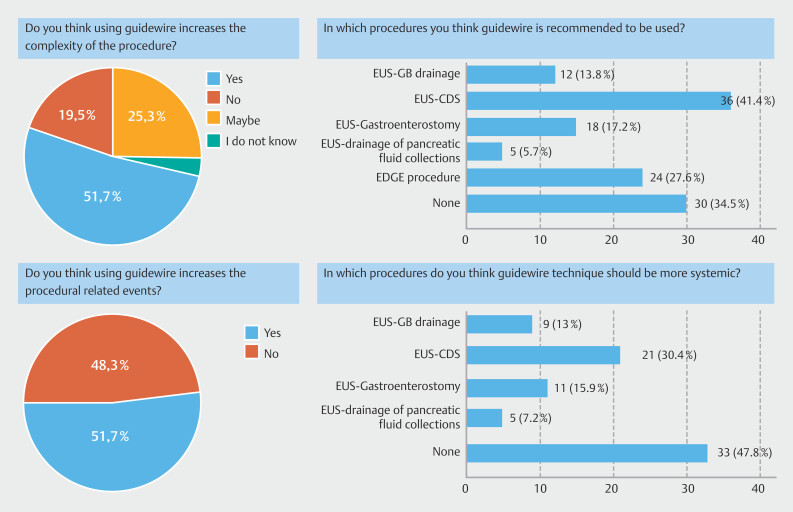
Perceptions of guidewire use in EC-LAMS procedures among the surveyed endoscopists and their recommendations on procedures where guidewire use should be considered.

Respondents were also asked to share their views on the role of guidewires during LAMS placement. Nearly half agreed that guidewire use increases procedure complexity and risk of AEs ( Fig. 2 ). Similarly, about 50% reported that guidewires should not be used systematically in electrocautery LAMS procedures ( Fig. 2 ). When asked for specific recommendations, 41.4% favored guidewire use in EUS-CDS and 27.6% in EDGE procedures, whereas only small minorities recommended routine use in EUS-GBD, PFC drainage, or EUS-GE. Regarding personal practice, 76.8% reported using guidewires in < 50% of cases, whereas 23.2% used them in > 50%. Among frequent users, the most common rationale was increased confidence (13% of all respondents). Additional reasons included training and habit (4.3%) and the perception that current evidence is insufficient to determine superiority of one approach (5.8%).

### Association of guidewire use with outcomes and impact of experience

Post-hoc analyses were performed to assess whether guidewire use correlated with AEs and whether operator experience influenced outcomes or technique choice ( Table 3 and Table 4 ). These analyses were based on aggregate self-reported experiences. Logistic regression analysis showed that use of a guidewire was associated with a higher LAMS-related AEs rate (20.8% vs. 17.8% in EUS-GBD, 44.1% vs. 20% in EUS-CDS, 20% vs. 9.3% in EUS-PFC, 17.9% vs. 14.6% in EUS-GE, and 55.6% vs. 28.3% in EDGE procedure). However, the higher rate was only statistically significant in EUS-CDS (OR 3.16, 95% CI 1.08–9.2, *P* = 0.03), whereas the rate did not achieve statistical significance in the other LAMS procedures ( Table 3 ). Moreover, there was no difference in the rate of overall complications among participants with < 5 years’ experience in EUS, EUS-guided tissue acquisition, and EUS-guided drainage procedures vs. 5 ≥ years ( Table 3 ). In addition, comparing EUS experience to < 5 years vs. ≥ 5 years, EUS-guided tissue acquisition, and EUS-guided drainage did not significantly influence guidewire usage.

**Table:** **Table 3** Association of guidewire use with LAMS maldeployment and overall AEs and effect of operator experience on AE rates.

	**EUS-GBD, n (%)**		**EUS-CDS, n (%)**		**EUS-PFC, n (%)**		**EUS-GE, n (%)**		**EDGE, n (%)**	
**Maldeployment**	**Yes**	**No**	**Yes**	**No**	**Yes**	**No**	**Yes**	**No**	**Yes**	**No**
**Guidewire use, N (%)**										
Yes	5 (20.8)	8 (17.8)	15 (44.1)	7 (20)	3 (20)	5 (9.3)	5 (17.9)	6 (14.6)	5 (55.6)	17 (28.3)
No	19 (79.2)	37 (82.2)	19 (55.9)	28 (80)	12 (80)	49 (90.7)	23 (82.1)	35 (85.4)	4 (44.4)	43 (71.7)
	OR 1.2, 95% CI 0.35–4.23, *P* = 0.76		OR 3.16, 95% CI 1.08–9.2, *P* = 0.03		OR 2.45, 95% CI 0.51–11.71, *P* = 0.26		OR 1.27, 95% CI 0.35–4.64, *P* = 0.72		OR 3.16, 95% CI 0.76–13.2, *P* = 0.11	
**Overall adverse events**										
**Experience in EUS**										
< 5 years	3 (10.7)	5 (12.2)	2 (5.1)	6 (20)	5 (17.9)	3 (7.3)	4 (11.8)	4 (11.4)	3 (18.7)	5 (9.4)
≥ 5 years	25 (89.3)	36 (87.8)	37 (94.9)	24 (80)	23 (82.1)	38 (92.7)	30 (88.2)	31 (88.6)	13 (81.3)	48 (90.6)
	OR 0.86, 95% CI 0.19–3.95, *P* = 0.85		OR 0.22, 95% CI 0.04–1.16, *P* = 0.07		OR 2.75, 95% CI 0.6–12.62, *P* = 0.19		OR 1.03, 95% CI 0.24–4.51, *P* = 0.96		OR 2.21, 95% CI 0.47–10.51	
**Experience in EUS-tissue acquisition**										
< 5 years	2 (7.1)	5 (12.2)	2 (5.1)	5 (16.7)	4 (14.3)	3 (7.3)	4 (11.8)	3 (8.6)	3 (18.7)	4 (7.5)
≥ 5 years	26 (92.9)	36 (87.8)	37 (94.9)	25 (83.3)	24 (85.7)	38 (92.7)	30 (88.2)	32 (91.4)	13 (81.3)	49 (92.5)
	OR 0.55, 95% CI 0.1–3.1, *P* = 0.5		OR 0.72, 95% CI 0.05–1.5, *P* = 0.13		OR 2.11, 95% CI 0.43–10.27, *P* = 0.35		OR 1.42, 95% CI 0.29–6.89, *P* = 0.66		OR 2.83, 95% CI 0.56–14.24, *P* = 0.21	
**Experience in EUS-guided drainage**										
< 5 years	5 (17.9)	12 (29.3)	7 (17.9)	10 (33.3)	5 (17.2)	12 (30)	7 (20.6)	10 (28.6)	3 (18.7)	14 (26.4)
≥ 5 years	23 (82.1)	29 (70.7)	32 (82.1)	20 (66.7)	24 (82.8)	28 (70)	27 (79.4)	25 (71.4)	13 (81.3)	39 (73.6)
	OR 0.53, 95% CI 0.16–1.7, *P* = 0.28		OR 0.44, 95% CI 0.14–1.33, *P* = 0.15		OR 0.49, 95% CI 0.15–1.58, *P* = 0.23		OR 0.65, 95% CI 0.21–1.96, *P* = 0.44		OR 0.64, 95% CI 0.16–2.59, *P* = 0.53	
AE, adverse event; CI, confidence interval; EDGE, EUS-directed trans-gastric endoscopic retrograde cholangiopancreatography; EUS-CDS, EUS-guided choledochoduodenostomy; EUS-GBD, EUS-guided gallbladder drainage; EUS-PFC, EUS-guided pancreatic fluid collection drainage; EUS-GE, EUS-guided gastroenterostomy; LAMS, lumen apposing metal stent; OR, odds ratio.										

**Table:** **Table 4** Association of EUS experience, annual case volume, and operator age with guidewire use during LAMS procedures.

	**EUS-GBD, n (%)**		**EUS-CDS, n (%)**		**EUS-PFC, n (%)**		**EUS-GE, n (%)**		**EDGE, n (%)**	
**Guidewire use**	**Yes**	**No**	**Yes**	**No**	**Yes**	**No**	**Yes**	**No**	**Yes**	**No**
**Experience in EUS**										
< 5 years	1 (7.7)	7 (12.5)	2 (9.1)	6 (12.8)	0	8 (13.1)	3 (27.3)	5 (8.6)	3 (14.3)	5 (10.4)
≥ 5 years	12 (92.3)	49 (87.5)	20 (90.9)	41 (87.2)	8 (100)	53 (86.9)	8 (72.7)	53 (91.4)	18 (85.7)	43 (89.6)
***P* value **	0.62		0.66		0.96		0.07		0.64	
**Experience in EUS-tissue acquisition**										
< 5 years	1 (7.7)	10 (17.8)	3 (13.6)	8 (17)	0	7 (11.5)	2 (18.2)	5 (8.6)	3 (13.6)	4 (8.5)
≥ 5 years	12 (92.3)	46 (82.2)	19 (86.4)	39 (83)	8 (100)	54 (88.5)	9 (81.8)	53 (91.4)	19 (86.4)	43 (91.5)
***P* value **	0.37		0.72		0.92		0.33		0.51	
**Experience in EUS-guided drainage**										
< 5 years	3 (23.1)	18 (32.1)	4 (18.2)	17 (36.2)	1 (12.5)	16 (26.2)	5 (45.5)	12 (20.7)	6 (27.3)	11 (23.4)
≥ 5 years	10 (67.9)	38 (67.9)	18 (81.8)	30 (63.8)	7 (87.5)	45 (73.8)	6 (55.5)	46 (79.3)	16 (72.7)	36 (76.6)
***P* value **	0.52		0.13		0.4		0.08		0.73	
**Number of cases/years**										
< 25 cases	3 (75)	71 (85.5)	16 (76.2)	56 (84.8)	2 (50)	59 (71.1)	13 (92.9)	61 (83.6)	27 (100)	56 (93.3)
≥ 25 cases	1 (25)	12 (14.5)	5 (23.8)	10 (15.2)	2 (50)	24 (28.9)	1 (7.1)	12 (16.4)	0	4 (6.7)
***P* value **	0.56		0.36		0.37		0.37		0.75	
**Age**										
≤ 40 years	4 (23.5)	27 (38.6)	6 (20)	24 (42.1)	3 (27.3)	27 (35.5)	6 (40)	24 (33.3)	9 (31)	21 (36.2)
> 40 years	13 (76.5)	43 (61.4)	24 (80)	33 (57.9)	8 (72.7)	49 (64.5)	9 (60)	48 (66.7)	20 (69)	37 (63.8)
***P* value **	0.25		0.04		0.59		0.62		0.63	
**Age**										
≤ 50 years	13 (72.2)	44 (63.8)	18 (60)	39 (68.4)	7 (63.6)	50 (65.8)	12 (80)	45 (62.5)	19 (65.5)	38 (65.5)
> 50 years	5 (27.8)	25 (36.2)	12 (40)	18 (31.6)	4 (36.4)	26 (34.2)	3 (20)	27 (37.5)	10 (34.5)	20 (34.5)
***P* value **	0.5		0.43		0.89		0.19		1	
**Age**										
≤ 60 years	14 (77.8)	64 (92.7)	25 (83.3)	53 (93)	9 (81.8)	69 (90.8)	14 (93.3)	64 (88.9)	26 (89.7)	52 (89.7)
> 60 years	4 (22.2)	5 (7.3)	5 (16.7)	4 (7)	2 (18.2)	7 (9.2)	1 (6.7)	8 (11.1)	3 (10.3)	6 (10.3)
***P* value **	0.06		0.16		0.36		0.61		1	
EDGE, EUS-directed trans-gastric endoscopic retrograde cholangiopancreatography; EUS-CDS, EUS-guided choledochoduodenostomy; EUS-GBD, EUS-guided gallbladder drainage; EUS-PFC, EUS-guided pancreatic fluid collection drainage; EUS-GE, EUS-guided gastroenterostomy; LAMS, lumen apposing metal stent.										

Similarly, annual procedure volume (< 25 vs. ≥ 25 cases per year) did not significantly influence guidewire use. A modest but statistically significant increase in guidewire utilization was observed among endoscopists aged > 40 years for EUS-CDS procedures because 38.6% of older respondents reported guidewire use compared with 23.5% of younger respondents ( *P* = 0.04), whereas no other age thresholds showed significant associations, although the overall trend was higher guidewire use among older practitioners ( Table 4 ).

## Discussion

This international survey offers a comprehensive overview of current practices in deployment of EC-LAMS, with a specific focus on guidewire usage. The key finding is that freehand deployment is the predominant technique across indications, whereas routine guidewire use is uncommon and generally reserved for challenging scenarios such as EUS-CDS and EDGE and seems to be favored by endoscopists aged > 40 years. This reflects a significant shift in practice compared with the pre-LAMS and “cold LAMS”-era, when guidewires were integral to EUS-guided stent placement 7 9 .

Our data may aid in clarifying current practice patterns regarding guidewire usage and illustrate that a guidewire may still be desirable in certain situations. For example, for EUS-CDS, the target (CBD) is mostly much smaller when compared to large fluid collections and misdeployment in this context may lead to potentially life-threatening complications such as biliary leak or peritonitis 12 13 14 . During EDGE, needle puncture of the excluded stomach to promote fluid injection is one fundamental step of the procedure, and therefore, that might lower the threshold for using a guidewire; moreover, scope stability and altered anatomy can make access more demanding, prompting some endoscopists to use a wire as a safeguard 15 16 . For both situations, guidewire use may outweigh increased procedure complexity. In contrast, for large pancreatic collections or gallbladder drainage, most practitioners found the freehand technique sufficient, while emphasizing the influence of target anatomy and scope positioning on decision-making.

Perceptions regarding guidewire use were divided. About half the respondents felt that wires add complexity and risk and nearly 50% advised against their routine use. Compared to a previous international Delphi consensus document on LAMS usage 17 , in the current manuscript, we also provide correlation with clinical outcomes: Guidewire use did not correlate with fewer AEs, in any indication. On the contrary, in EUS-CDS, guidewire use was reported to be associated with a significantly higher complication rate, particularly maldeployment. Several interpretations are possible. The guidewire itself may contribute to technical difficulties because its presence can alter the axis of the delivery system or change scope torque, thereby hindering controlled stent release. An imperfectly positioned wire may also create a false sense of security, prompt advancement of the catheter beyond the optimal depth, or direct endoscopist attention away from the sonographic view. An alternative explanation is selection bias because guidewires are more often used in technically demanding cases or may be preferred as a lifeline by those who have experienced AEs during that procedure. These points suggest that a guidewire does not necessarily prevent technical failure and that selecting a CBD diameter > 15 mm is key in the context of EUS-CDS 18 .

Complication patterns observed in the survey are consistent with clinical experience.

Maldeployment was the most frequently encountered issue across procedures, particularly in EUS-CDS and EUS-GE, underscoring the need to master every single step during LAMS placement. Maldeployment can lead to severe consequences, including peritoneal leakage, bleeding, or need for emergency surgery 6 19 . Preventing this complication goes beyond LAMS insertion itself and should include meticulous endosonographic assessment of the target, trajectory selection, scope stabilization, as well as precise deployment technique and controlled retraction of the echoendoscope during flange release 3 20 . Although a guidewire could theoretically assist in LAMS re-access, the EUS tract is often lost when maldeployment occurs due to the extraluminal location of one of both flanges 21 22 . The added benefit of a safety guidewire should carefully be weighed against the increased complexity, especially because use of a guidewire forces a specific LAMS advancement direction, which may not imply the best operative window for LAMS release; furthermore, advancement of the guidewire itself might push a mobile target away and cause loss of acoustic coupling. Looking at other reported complications, bleeding was perceived as the most common AE in PFC drainage, aligning with previous data in the context of walled-off necrosis 23 . EDGE, on the other hand, was associated with comparatively fewer reported complications, likely reflecting careful patient selection and concentration of cases among highly experienced endoscopists.

Operator factors were also explored. Interestingly, less experienced endoscopists did not report higher complication rates or greater reliance on guidewires than their senior colleagues, suggesting that EC-LAMS technology, combined with training and mentorship, seem to allow for safe adoption even early in the learning curve. Several factors may explain this finding. Electrocautery-enhanced stents have simplified the procedure, reducing the number of steps and thereby limiting opportunities for error, which may narrow the gap between trainees and experts 24 . However, age seemed to influence practice patterns: older endoscopists, trained in the pre-EC-LAMS era, were more likely to use guidewires, reflecting a generational difference in technique preference.

These findings may have several implications for our everyday practice. First, freehand deployment is regarded as safe and effective for most LAMS indications and has been adopted by endoscopists regardless of career stage, taking into account appropriate training and proctoring. Second, our analysis shows that guidewire usage may be considered selectively, such as in the context of EUS-CDS and challenging EDGE cases. Third, training programs should emphasize proficiency with freehand techniques while also teaching judicious use of wires when warranted.

Although data focused on current use of guidewire during LAMS procedures are extremely scarce, our study also has limitations. As a self-reported study, responses are subject to recall bias and variation in definitions (e.g., what constitutes a “maldeployment”). The sample, although international, was relatively small and enriched for high-volume experienced LAMS users, which raises questions regarding generalizability. Another limitation is the comparison of self-reported complication rates between those who use a guidewire and those who do not. However, self-reported outcomes, while inherently limited, provide insight into operator experience, perceived safety, and technique-related concerns that are difficult to capture in controlled trials, particularly for evolving procedures such as LAMS deployment, because the objective of comparing complication rates between guidewire and non-guidewire techniques was not to determine causality or precise procedure risk, but rather, to explore real-world practice patterns and perceived safety among endoscopists. The study also is limited by different participating endoscopist geographic location, procedure volume, experience, and case mix which preclude adjustment for operator experience, procedure complexity, and learning-curve effects, which therefore prevent causal or risk-based comparisons. Assessment of respondents having at least one case of each AE also is a limitation **.** We acknowledge that occurrence of AEs in interventional endoscopy is, to a large extent, time- and exposure-dependent. Accordingly, a negative response does not imply absence of risk but may reflect limited cumulative procedural volume or shorter operator experience. This variable was included to capture prior personal exposure to specific complications rather than to estimate true incidence or procedural safety. Finally, our study was not designed to define definitive procedure indications for guidewire use or to establish objective safety outcomes. Rather, the primary aim was to explore expert perceptions and practice patterns regarding guidewire use in specific clinical and technical contexts. Our findings do not seek to imply causality or confirm procedure safety, but instead, to identify factors that experts associate with a perceived increase in safety.

Despite these limitations, this study is, to our knowledge, among the first to systematically explore guidewire use in EC-LAMS procedures.

This survey highlights the need for further research. A logical next step would be a prospective study or multicenter registry comparing outcomes of LAMS procedures performed with and without guidewire assistance, to clarify whether one approach confers greater safety or success ideally stratified by anatomical and procedure complexity. Specific questions, such as whether guidewires reduce malemployment in small ducts or, instead, contribute to it, could be addressed with larger datasets. If robust evidence confirms no benefit, device instructions may increasingly recommend the freehand technique to support its routine adoption.

## Conclusions

Freehand insertion has emerged as the predominant technique across indications. Routine guidewire use was not perceived to reduce procedure risks and outcomes as expressed by the operators, although older endoscopists were more likely to use guidewires, reflecting generational training differences.

As LAMS technology continues to evolve, sharing expert practices can help refine training and inform the development of future prospective studies to establish evidence-based best practice.

## Supplementary Material

Supplementary Material Zusatzmaterial
