# Supplementary material for: Guidewire use in electrocautery-enhanced lumen-apposing metal stent procedures: Results of an online survey from an international working group
Source: Endosc Int Open. 2026 May 20;14:a28547045. doi: 10.1055/a-2854-7045 (PMC13289957; doi:10.1055/a-2854-7045)

Supplementary table 1 Number (%) of participants who reported LAMS procedure-related adverse events.

	Number of participants who reported no adverse events	Number of participants who reported bleeding	Number of participants who reported perforation	Number of participants who reported maldeplyoment	Number of participants who reported other adverse events	Number of participants who answered the question
**EUS-GBD**	40 (58%)	5 (7.2%)	4 (5.8%)	24 (34.8%)	4 (5.8%)	69
**EUS-CDS**	28 (40.6%)	11 (15.9%)	4 (5.8%)	34 (49.3%)	2 (2.9%)	69
**EUS-PFC**	39 (56.5%)	18 (26.1%)	2 (2.9%)	13 (18.8%)	2 (2.9%)	69
**EUS-GE**	33 (47.8%)	3 (4.3%)	8 (11.6%)	28 (40.6%)	3 (4.3%)	69
**EDGE**	47 (68.1%)	5 (7.2%)	4 (5.8%)	9 (13%)	6 (8.7%)	69

EDGE, endoscopic ultrasound-directed transgastric endoscopic retrograde cholangiopancreatography; EUS-CDS, EUS-guided choledochoduodenostomy; EUS-GBD, EUS-guided gallbladder drainage; EUS-GE, EUS-guided gastroenterostomy; EUS-PFC, EUS-guided pancreatic fluid collection drainage; LAMS, lumen apposing metal stent.

Supplementary figure 1. Participants countries of origin.

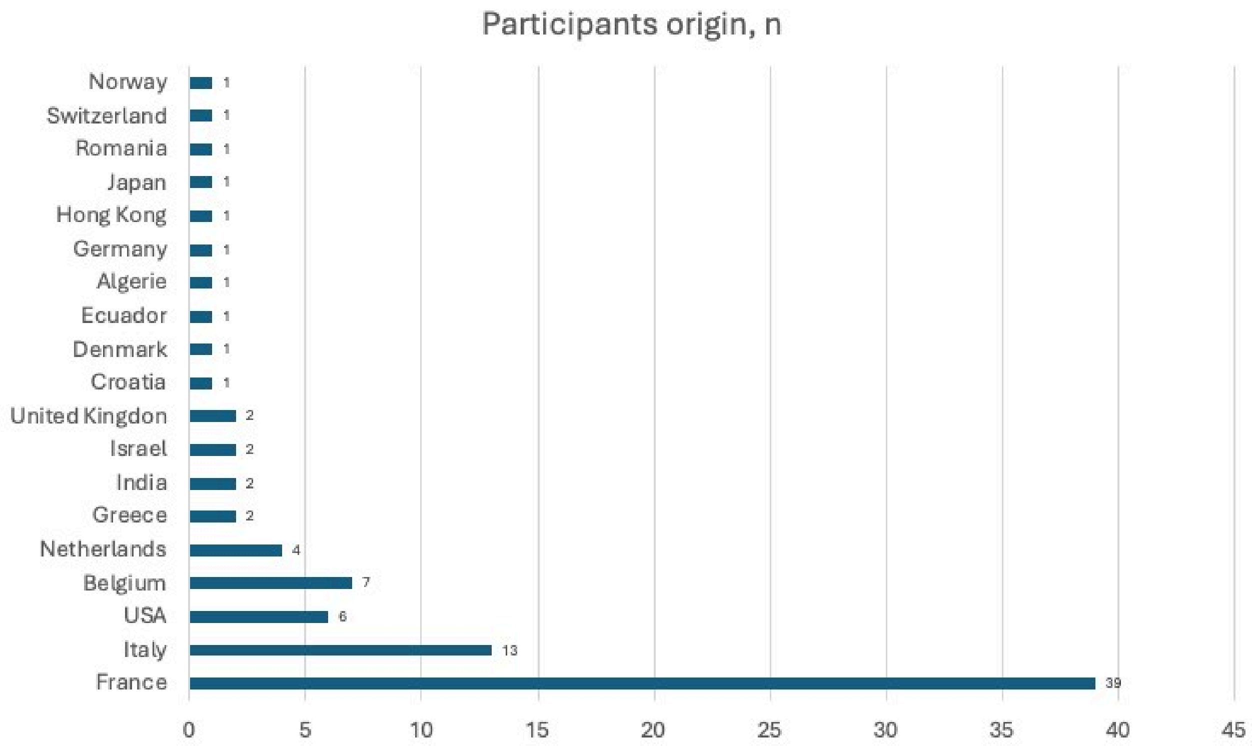

Supplement: Supplementary file 1 — Supplementary Material [file 10-1055-a-2854-7045_28580438.pdf]
